# Acute exposure to diesel exhaust induces central nervous system stress and altered learning and memory in honey bees

**DOI:** 10.1038/s41598-019-41876-w

**Published:** 2019-04-08

**Authors:** Christine M. Reitmayer, James M. W. Ryalls, Emily Farthing, Christopher W. Jackson, Robbie D. Girling, Tracey A. Newman

**Affiliations:** 1 CES, Faculty of Medicine, Institute for Life Sciences, M55, University of Southampton, Southampton, SO17 1BJ UK; 2 School of Agriculture, Policy and Development, University of Reading, Reading, RG6 6AR UK; 3 School of Biological Sciences, Life Sciences Building 85, University of Southampton, Southampton, SO17 1BJ UK

## Abstract

For effective foraging, many insect pollinators rely on the ability to learn and recall floral odours, behaviours that are associated with a complex suite of cellular processes. Here, we investigated how acute exposure to a high-dose of diesel exhaust (containing 19.8 and 17.5 ppm of NO and NO_2_, respectively) affected associative learning behaviour of honey bees (*Apis mellifera*) and expression of a ubiquitous heat shock protein, HSP70, in their central nervous system (CNS). To determine whether exposure to diesel exhaust would alter their tolerance to a subsequent abiotic stress, we further subjected individuals to heat stress. Diesel exhaust exposure decreased honey bees’ ability to learn and recall a conditioned odour stimulus. Whilst there was no significant difference in CNS HSP70 expression between honey bees exposed to either diesel exhaust or clean air across the entire duration of the experiment (3.5 h), there was a significant effect of time and a significant interaction between exposure treatment and time. This interaction was investigated using correlation analyses, which demonstrated that only in the diesel exhaust exposed honey bees was there a significant positive correlation between HSP70 expression and time. Furthermore, there was a 44% reduction in honey bee individuals that were able to recall the odour 72 h after diesel exposure compared with clean air control individuals. Moreover, diesel exhaust affected *A. mellifera* in a way that reduced their ability to survive a second subsequent stressor. Such negative effects of air pollution on learning, recall, and stress tolerance has potential to reduce foraging efficiency and pollination success of individual honey bees.

## Introduction

Pollination services are critical for production of many crops with, for example, 84% of crop species cultivated in Europe benefitting from insect pollination^1,2^. Therefore, maintaining healthy pollination services is an essential component of ensuring future global food security^3^. However, declines in wild and managed pollinators, linked to climate change, pesticide use, pathogens, invasive species, and changes in land-use are occurring across multiple regions of the world^4^. Recent studies have demonstrated that air pollution may also be contributing to disruption of pollination services by degrading floral scents that insects require to locate flowers^5,6^.

Nitrogen oxide (NOx) gases released in diesel exhaust, for example, can alter the chemical composition of floral volatiles, which, in turn, affects honey bees’ (*Apis mellifera*) ability to recognise an altered odour^7,8^. This species is the most widespread managed pollinator and despite a global increase in numbers of managed colonies (ca. 45%) compared with five decades ago, large-scale seasonal losses have been reported within the last decade^9,10^. Considering the importance of odour communication for pollinators in foraging for floral resources (i.e. nectar and pollen), this change in floral recognition could lead to declines in pollinator fitness and foraging efficiency^11^.

Beyond disruption of floral scents, diesel air pollution is more widely reported for its direct negative effects on mammalian health, including the cardiovascular system and the central nervous system (CNS)^12,13^. However, we understand very little about direct effects that diesel exhaust pollution has on insect health and behaviour. Diesel exhaust pollution includes a range of highly reactive constituents, which could potentially interfere with receptor cells on an insect’s antennae or enter an insect through its spiracles. Only a handful of studies have demonstrated direct effects of air pollutants on insects in general^14^ with, for example, SO_2_ exposure reducing brood rearing and flight activity of male sweat bees (*Lasioglossum zephyrum*) and honey bees^15,16^. The effects of pollutants on insects tend to be plant-mediated; however, direct effects can be toxic, metabolically stimulating or behaviourally altering (see^17^ and references therein).

The aim of our current study was to determine whether acute exposure to diesel exhaust pollution interferes with associative learning and memory formation, essential functions of a honey bee’s CNS. Effective foraging is the basis of pollinator success, requiring complex cognitive abilities associated with decision making, learning, memory, and communication^18^. Any degradation of these abilities is likely to have negative impacts upon pollinator foraging and success. The complex social, navigational, and communication behaviours of honey bees and relative simplicity of their well-studied CNS make them excellent models for testing learning and memory function in response to atmospheric changes^19,20^.

Diesel exhaust emission is a complex mixture of organic and inorganic compounds in both gaseous and particulate form. Many of these components have the potential to affect cells within the structures of the honey bee brain which are crucial for associative learning of odour and reward. In response to stressful conditions, cells undergo a range of molecular changes to protect themselves against detrimental influences, minimise cellular damage, and ideally re-establish homeostatic control^21^. One consequence of cellular stress is accumulation of unfolded or misfolded proteins in cells. Molecular chaperones assist with protein folding, transport and, if necessary, degradation. In cases where damage to a protein is substantive, such as misfolding, degradation of the misfolded protein is induced^22^. Many heat shock proteins (HSPs) are up-regulated by a variety of stressors, including heat^23^, anoxia^24^, heavy metals^25^, UV radiation^26^, and pesticides^27^. Diesel inhalation by mice can induce one of the largest stress protein families, HSP70^28^. Upregulation of HSP70 also occurs in honey bee brain tissue in response to ethanol exposure^29^, and such exposure can impair honey bee associative learning behaviour^30^. It could therefore be hypothesised that changes in associative learning and memory recall of honey bees in response to diesel pollution could be associated with changes in the regulation of HSP70. Moreover, physiological responses of honey bees and other insects to air pollution may have direct negative (e.g. decreased immune function) but non-lethal effects on an individual, which may reduce its ability to cope with additional abiotic stress factors^31,32^.

By subjecting honey bees to an acute exposure of diesel exhaust, this study assessed their ability to learn and memorise a floral odour. Throughout this study we use the definitions provided by Frost *et al*.^33^ for these terms, i.e. “learn” means that honey bees have shown evidence of associating a conditioned stimulus with a reward and “memorise” means they have shown evidence of storing and then retrieving that association after a range of defined time intervals. It was hypothesised that associative learning performance of honey bees would decrease, concomitant with an upregulation of HSP70. HSP70 is a central protein in the heat shock response cascade; it is a prosurvival response (in contrast to cell-death responses) intended to re-establish homeostasis^34,35^. HSP70 protein expression was used as a marker for a cellular stress response in the brain due to its broad distribution, its responsiveness to a broad variety of stressors (in contrast to constitutively expressed members of the HSP family) and its central role within the HSP response^36^. In addition, heat stress after exposure to diesel was used to determine whether diesel pollution would alter the vulnerability of honey bees to a second environmental stressor. We hypothesised that honey bees exposed to a heat stress event after acute exposure to diesel pollution would have a reduced rate of survival in comparison to individuals that were not exposed to diesel exhaust pollution.

## Results

### Learning performance and memory retrieval

**Figure 1:**
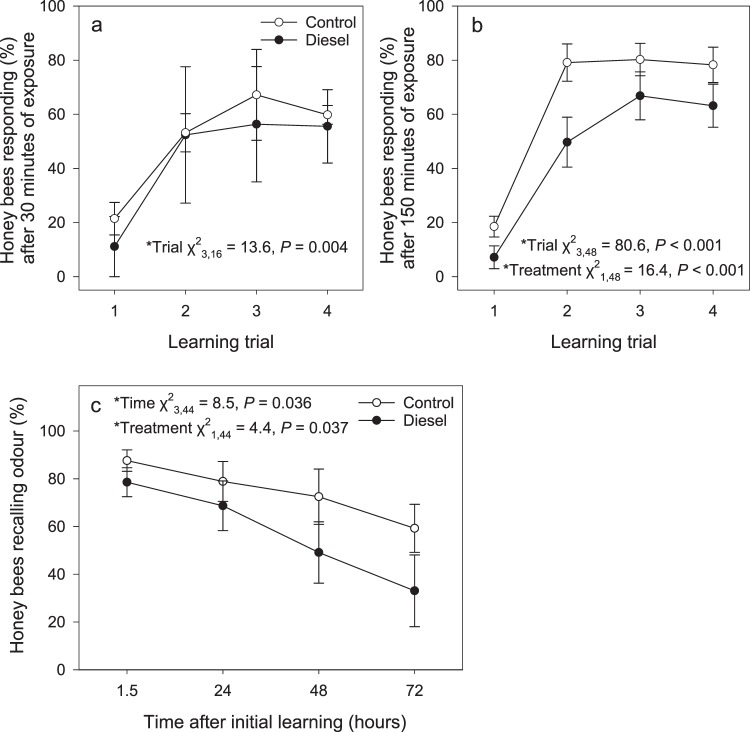
The percentage of honey bees (*Apis mellifera*) that responded to the conditioned stimulus (linalool) with a proboscis extension response within the first 10 s of odour presentation in four learning trials after 30 min of air treatment exposure (N_control_ = 22, N_diesel_ = 20) (**a**) and in four learning trials after 150 min of air treatment exposure (N_control_ = 66, N_diesel_ = 65) (**b**). For the honey bees that were given a 150-minute air treatment exposure and that exhibited a conditioned response during the learning trials, subsequent assessments were made to assess their ability to recall the conditioned odour information. It was recorded whether honey bees extended their proboscis in response to the conditioned stimuli at four different time points (1.5, 24, 48, 72 h) after the initial conditioning trials (N_control 1.5,24,48,72h_ = 59, 55, 47, 29; N_diesel 1.5,24,48,72h_ = 47, 42, 37, 23) (**c**). Values displayed are means (±SE). Significant beta regression model statistics shown. Full model statistics shown in Table S1.

All honey bees used in learning and memory retrieval experiments were initially exposed to either a diesel exhaust or a clean air treatment and then assessed to ensure they could extend their proboscis in response to sucrose solution, with only responsive animals used in subsequent tests. A 30-minute exposure to a high-dose of diesel exhaust (full concentrations and constituents as described in Girling *et al*.^7^; briefly, levels were ca. 19.8 and 17.5 ppm of NO and NO_2_ respectively) did not alter the ability of honey bees to learn a conditioned odour stimulus (linalool) in comparison to the control group (Fig. 1a; Table S1). Honey bees exposed to diesel exhaust for 150 minutes demonstrated a significant reduction in their ability to learn linalool over the course of the four learning trials in comparison to the control group (Fig. 1b; Table S1). Those honey bees treated for 150 minutes that responded positively to the conditioned odour, i.e. that had learnt the odour, were subsequently tested to assess their ability to recall this odour. The proportion of honey bees that could recall linalool significantly decreased with time in both diesel exposed and control groups, but there was also a significant effect of treatment, with honey bees in the diesel exhaust exposure treatment group less able to recall the odour (Fig. 1c; Table S1).

### HSP70 protein expression in CNS tissue

**Figure 2:**
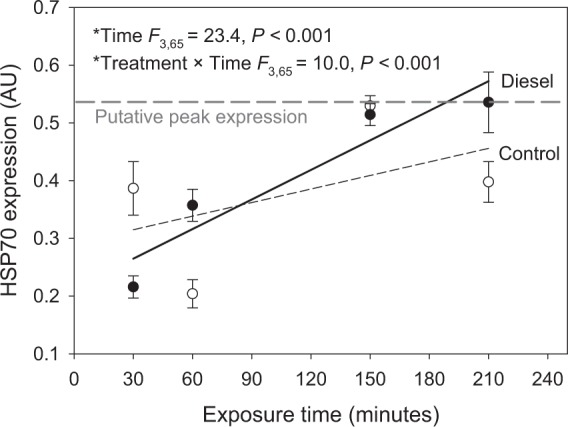
The effects of diesel exhaust exposure versus a clean air control exposure on HSP70 protein expression in the brain of honey bees (*Apis mellifera*). AU refers to arbitrary units of HSP70 normalised against β-tubulin. N_control_ = 34, N_diesel_ = 40. Values displayed are means (±SE). Significant linear model statistics shown. Full model statistics shown in Table S1.

Comparing HSP70 expression between honey bees exposed to either diesel exhaust or clean air treatments across the entire duration of the experiment (3.5 h) demonstrated no significant effect of treatment (Table S1); however, there was a significant effect of time (Fig. 2); and a significant interaction between exposure treatment and time (Fig. 2). Further investigation of this interaction using correlation analyses demonstrated that when honey bees were not exposed to diesel exhaust, HSP70 protein expression fluctuated over time, with no significant correlation between exposure time and HSP70 protein expression (Fig. 2; df = 31, *r* = 0.24, *P* = 0.17). In individuals exposed to diesel exhaust, HSP70 protein expression generally increased as diesel exhaust exposure time increased (df = 38, *r* = 0.77, *P* < 0.001). HSP70 can be upregulated by a variety of stressors and, therefore, in investigations with free living organisms, such as honey bees, some additional variation between time points and treatment groups is expected. As such, it is more informative to observe the broader trends in expression over time points and comparisons of individual time points should be made with caution.

Whilst acknowledging this caveat, HSP70 protein expression in diesel-exposed individuals was lower, higher, and higher after 30, 60, and 210 minutes, respectively, compared with control individuals. HSP70 expression did not vary between exposed and control individuals after 150 minutes. In addition, standard errors for both treatments reduced at this time point indicating that HSP70 protein expression may have reached a “peak” value. After 210 minutes, HSP70 protein expression in diesel exposed honey bees remains around this “peak” level; however, HSP70 protein expression in clean air control bees decreased back to similar levels that were recorded after a 30-minute exposure, despite the fact that the 210-minute treatment was seven times as long.

### Survival and resistance to heat stress after acute diesel exhaust exposure

**Figure 3:**
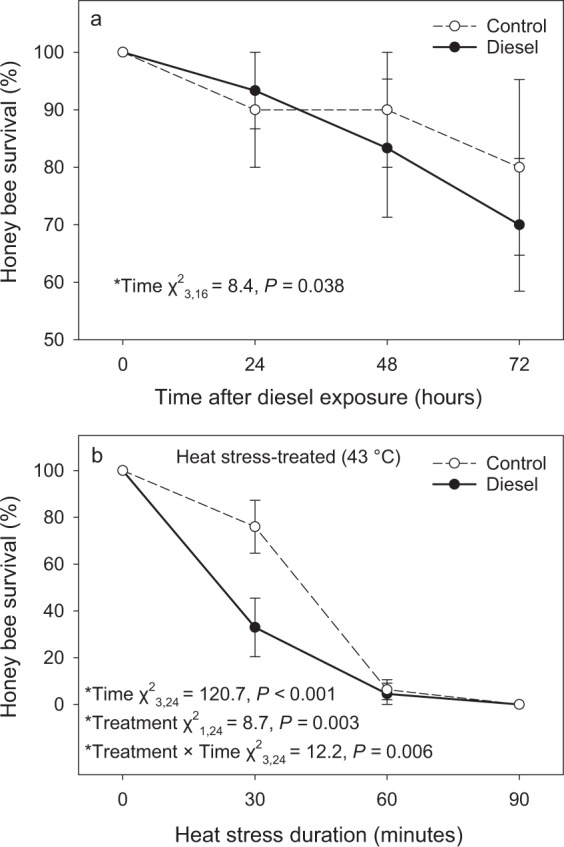
The effects of a diesel exhaust exposure versus a clean air control exposure (150 min) on the survival of honey bees (*Apis mellifera*) over 72 h (N_control_ = 12, N_diesel_ = 12) (**a**) and the effects of a diesel exhaust exposure versus a clean air control exposure (150 min), followed by a heat stress event (43 °C), on the survival of honey bees over 90 min (N_control_ = 16, N_diesel_ = 16) (**b**). Values displayed are means (±SE). Significant beta regression model statistics shown.

Honey bee survival decreased over time (survival at 0 h was significantly higher than survival at 72 h) but diesel exhaust exposure had no effect on honey bee survival versus the clean air control (Fig. 3a; Table S1). For honey bees that were subjected to a heat stress after an exposure to either diesel exhaust or a clean air control, air treatment and time interacted to affect honey bees’ survival. Thirty minutes of heat stress, in particular, resulted in a 57% decrease in survival for bees that were previously exposed to diesel exhaust compared to honey bees from the control air treatment group (Fig. 3b; Table S1).

## Discussion

Foraging success of pollinators, underpinned by associative learning (i.e. associating a floral odour with a nectar reward), is highly dependent on their ability to memorise and recall a plant’s floral odour blend^37^. Acute exposure to a high-dose of diesel exhaust had clear effects on associative learning behaviour and stress response of *A. mellifera*. In particular, diesel exhaust exposure significantly decreased the ability of *A. mellifera* to learn the conditioned stimulus and reduced their ability to remember and respond to that odour over a 72-hour period compared with individuals that were not exposed to diesel exhaust. Whereas 30 minutes of exposure to diesel exhaust did not affect the response of *A. mellifera*, 150 minutes of exposure reduced learning behaviour. Moreover, as time progressed (i.e. by 72 h after initial conditioning), exposure to diesel exhaust almost halved the proportion of individuals that were able to recall the odour stimulus compared with clean air control individuals. These results indicate that individuals exposed to diesel exhaust are likely to perform worse in learning to associate a floral volatile with a nectar reward, memorise this information and recall it later. Longer foraging bouts or colony establishment in high pollution areas therefore has the potential to result in deleterious effects on honey bee foraging success. Previous work on honey bees has demonstrated that floral volatiles are degraded by reactive components of diesel exhaust, which can subsequently affect the ability of honey bees to recognise an odour blend^7,8^. Therefore, the combined effect of impaired learning and memory and an altered olfactory environment has potential to impair foraging efficiency of pollinators. However, honey bees do not rely solely on olfactory cues to find and recognise known foraging sites but also on visual aids, such as landmarks^38^. It is therefore possible that air pollution could increase dependence of pollinators on visual cues for successful foraging, as previously suggested by Girling *et al*.^7^

It is not possible from the current work to conclusively link changes in expression of HSP70 protein with changes observed in learning and memory response. We can only state that there was a significant positive correlation between duration of exposure to diesel exhaust and HSP70 expression in the CNS of *A. mellifera* and that such an exposure also resulted in a reduction in their capacity to learn or recall a floral volatile odour. Associative conditioning, required for the proboscis extension response (PER) assay used in this work, can establish different types of memories^39,40^. A short-term memory of the association between an odour and a reward can be achieved after only one or a few conditioning trials. Results from the PER assays demonstrated that honey bees treated with a 150-minute exposure to diesel exhaust had a functional, although somewhat degraded, short-term memory, but a more significantly impaired long-term memory, which is suggestive of changes associated with altered protein expression in the brain. Short-term memory formation is considered to be independent of protein synthesis, whereas long-term memory requires mRNA transcription and subsequent protein synthesis^41^. Expression of HSP70 protein had no clear pattern under ambient conditions; however, there was a significant correlation between HSP70 expression and duration of exposure to diesel exhaust. The design of the behavioural and molecular analyses resulted in a difference in timing between the end of the exposure treatment and the exact time that the analyses were conducted for the PER and HSP70 expression assays. Therefore, it is not possible to directly compare results for PER learning, memory, and HSP70 expression analysis after the same treatment duration. HSP70 is a marker of a cellular stress response and as such expression can be influenced by a variety of factors. The culmination of this response is a return to a homeostatic baseline. How quickly the return to a homeostatic state is achieved after the end of exposure to a stressor varies by species, tissue type exposed, duration, and nature of the stressor^34,35^. This response in honey bees acutely exposed to diesel exhaust has not been studied previously. There is some variability in expression between individual time points, which can be expected due to the ubiquitous nature of the response of HSP70 to cellular stress. However, a clear picture emerges when observing the results over the duration of our experiment; this reveals large variations in HSP70 expression in the CNS of control bees and, in contrast, a steady increase of HSP70 expression in diesel exhaust exposed bees positively linked to exposure duration. This is an important and relevant finding; in nature, insects are exposed to a variety of environmental stressors that may counteract or exacerbate each other^42,43^. Despite not being able to identify a definitive mechanism from our study to explain the HSP70 findings, it highlights the variable baseline in expression between animals which in turn is likely predictive of outcomes. It should encourage further investigation into the relationship between HSP70 protein expression and exposure to diesel exhaust.

In our study, honey bees exposed to diesel exhaust prior to heat stress had a significantly higher mortality rate 30 minutes into a heat exposure compared with individuals from the control group. This further suggests that diesel exposure may be disrupting cellular stress machinery in *A. mellifera*, with knock-on effects on tolerance to additional stress factors. Studies combining air pollution and pollinator responses under natural field conditions would therefore provide a significant step towards understanding ecological effects of air pollution on pollinators and their interactions with other biotic and abiotic factors.

Ultimately, these data demonstrate the detrimental effects of air pollution on the learning behaviour of honey bees and their tolerance to additional stress factors. These negative effects may well extend beyond honey bees to other pollinators and insect guilds. In fact, those that rely more heavily on odour cues over visual cues, such as bumble bees^44^, may suffer more acutely from exposure to such pollution. With the threats of climate and atmospheric change on pollinator communities apparent, combining multiple environmental factors in ecological studies is key to maintaining ecosystem services such as pollination, and securing our food resources, especially as human population levels and agricultural demand continue to increase^45^.

## Materials and Methods

### Honey bee maintenance and collection

Honey bees were kept on the University of Southampton campus in an apiary (50°56′10″N, 1°23′39″W) containing four Langstroth hives, each with one brood box and 1–2 super boxes. Hive checks were carried out weekly, during which queen cells were removed; a *Varroa* screen was installed on all hives and checked weekly to control for *Varroa destructor* infestation. Hive checks were performed without the use of smokers; hives were not treated against *Varroa* or *Nosema*. Yearly FERA inspections took place in the apiary. For all assays, returning pollen foragers were collected at the hive entrance in the late morning hours (between ten am and twelve noon). For each individual experiment all bees were sourced from a single hive. Honey bees were caught using Sterilin 30-ml universal containers (Sterilin Limited, Cambridge, UK) and transferred into 32.5-cm^3^ polyester mesh insect cages (BugDorm, Taiwan) ready for use in assays.

### Acute exposure to diesel exhaust

Honey bees were placed into one of two ca. 3.5-litre glass exposure chambers into which either filtered clean air (control) or a diesel exhaust mix were pumped via Teflon tubing. For each experimental replicate, ca. ten honey bees per treatment were transferred from the mesh cages immediately following collection and randomly assigned to the control or diesel exposure chambers. Honey bees were supplied with 30% sucrose solution (in dH_2_O) *ad libitum*.

Air supplied to control chambers, was passed through a charcoal filter and humidified by bubbling through a distilled water trap (Fig. S1). The distilled water trap was installed to re-humidify air which would have been stripped by the charcoal filter. In addition, approximately 11% of diesel exhaust gases are water^46^; therefore the water trap also ensured that humidity was added to both control and diesel air flows. Exhaust supplied to diesel chambers was generated using a Suntom SDE 6500 E diesel generator (Fuzhou Suntom Power Machinery Co., Ltd. Fuzhou, China) which was started and run for 30 min before exposures to establish a steady running temperature and exhaust emissions. Silicon tubing (50 cm × 3 cm external diameter) was attached to the generator’s exhaust and a smaller diameter silicon tube (4 m × 8 mm external diameter, Fisher) placed inside this to transfer exhaust gases, via a pump, into the glass diesel chamber. Air flow into both diesel and control chambers was regulated to 1 L/min using inline flow-meters. Chambers were placed in a water bath to maintain a consistent internal temperature, which was measured at between 20–25 °C. The duration of exposures was either 30, 60, 150 or 210 min as stated.

Honey bees treated for use in PER assays and survival experiments were transferred from exposure chambers back into 32.5 cm^3^ polyester mesh insect cages for immediate transport to the laboratory. Honey bees treated for use in molecular analyses were immobilized by placing the chambers into ice water then removed from chambers and decapitated. A small incision was made in the eye region (taking care not to damage brain structures) to allow penetration of RNAlater (Ambion) and heads were stored in Eppendorf tubes for subsequent dissection and protein analyses.

### Proboscis Extension Response assay

Assays were conducted in an air-conditioned room at ca. 20–23 °C, lit by both natural (through windows) and artificial light (commercially available ceiling lightning). Honey bees were immobilized by cooling individually on ice in 50 ml falcon tubes before being transferred and harnessed into PER testing tubes (see^7^ for further details). Only honey bees showing an initial PER elicited by 30% sucrose solution were used.

Each honey bee was trained to associatively learn the synthetic floral volatile linalool using the method described in Girling *et al*.^7^ and Lusebrink *et al*.^8^. Linalool was selected because it elicits a strong PER in honey bees^47^. Individual restrained honey bees were placed into a well-ventilated PER chamber (which eliminated other sources of visual and odour stimuli). After 10 s of acclimatization, the honey bee was exposed to an odour stream from a glass tube containing a filter paper impregnated with 10 μl of linalool for 10 s. Five seconds into the odour stimulus the honey bees’ antennae were touched with a wooden toothpick carrying a droplet of 30% sucrose solution and honey bees were allowed to feed for 10 s. The test chamber was vented for 30-s before the subsequent honey bee was introduced. Honey bees were scored as having learnt the conditioned stimulus (linalool) if a proboscis extension was observed after onset of the odour stimulus and before the application of the unconditioned stimulus (sucrose). The learning trial was repeated four times for each honey bee, which was demonstrated to be sufficient for learning to occur in preliminary trials and is in line with recommendations in the literature^48,49^. The inter-trial interval for each honey bee was 10 min. Whilst opinions in the literature are divided over the most suitable inter-trial interval length, ranging from 5 to 30 min^33^, 10 min has been shown to facilitate the production of a robust and stable long-term memory^39^. Any honey bees that performed a proboscis extension in response to air flow alone when first placed in the PER chamber, in the absence of linalool, were excluded from trials.

To test each honey bee’s ability to memorise the association formed during the learning trial, a memory retrieval test was conducted for honey bees given a 150-min exposure period. For this, the PER assay was repeated at 1.5 h, 24 h, 48 h, and 72 h after initial conditioning. Each honey bee was tested once at each memory retrieval time point and no reward was given. Between memory tests, honey bees were kept restrained in a room (ca. 20–23 °C) separate from the PER testing apparatus and fed twice a day with 30% sucrose solution.

### HSP70 expression

HSP70 protein expression was measured from control and diesel treated honey bees that were exposed for 30, 60, 150, and 210 min. Complete brains were dissected out of the head capsule in phosphate-buffered saline (PBS) solution and homogenized in lysis buffer (Tris (pH 7.4) 50 mM, Sodium chloride 150 mM, NP40 1%, complete protease inhibitor (Roche) according to manufacturer’s instructions) using a probe sonicator.

Quantification of protein content was determined against a bovine serum albumin (BSA) standard curve (2 mg/ml – 0.03 mg/ml). Protein samples were separated using SDS-Poly-Acrylamide gel electrophoresis (PAGE) gels (BioRad mini protein II) using a 12.5% acrylamide resolving gel. Gels were run at 50 mA for migration through the resolving gel with each protein sample run in duplicate.

Proteins were electro-transferred from SDS-PAGE gels onto a nitrocellulose membrane at 4 °C at 30 V for 17 h. After transfer, immobilized proteins were incubated in 5% non-fat milk powder solution (5% w/v in PBS/0.1% Tween-20). Membranes were cut at the respective position, followed by an incubation overnight at 4 °C with 2.5% non-fat milk powder solution (2.5% w/v in PBS/0.1% Tween-20) containing one of the following primary antibodies: anti-HSP70 monoclonal antibody (Sigma, 1:1000) or anti-β-tubulin monoclonal antibody (Hybridoma Bank, 1:1000). Membranes were washed in PBS/0.1% Tween-20 and incubated for 1 h at room temperature in the dark with fluorescently labelled secondary antibody (anti-mouse Alexa Fluor 800 nm (Thermo-Fisher), 1:10000).

Protein band intensities were analysed using a LiCor Odyssey infrared detection system at 700 nm and 800 nm wavelengths, following the D_c_ protein assay method as described in the manufacturer’s instructions. Fluorescence intensity associated with specific immuno-reactivity was measured and expression of HSP70 protein was normalized against β-tubulin protein expression.

### Survival after exposure

To test if acute exposure to diesel exhaust affected survival of bees, individuals were observed for two days and their survival was recorded. All animals were exposed to either diesel exhaust or control conditions as previously described and placed into 32.5 cm^3^ polyester mesh insect cages after the end of the exposure. The exposure time was 150 min. The animals were kept in the insect cages in the invertebrate facility laboratory under controlled temperature and humidity. Food was supplied ad libitum in the form of 30% sucrose in dH_2_O. Survival was recorded 24 h and 48 h after the acute exposure. Three exposures each with 10 animals per exposure group were carried out.

### Secondary, abiotic, stress resistance

Honey bees possess a relatively high tolerance to a short period of high temperature. To investigate whether acute exposure to diesel exhaust affects this ability to tolerate heat stress, honey bees were exposed to 43 °C and survival was monitored every 30 min. Honey bees were treated with diesel exhaust and control conditions for 150 min as described above and subsequently transferred into vials with a diameter of 2.5 cm and a height of 8 cm. The vials were closed using a moist sponge, which ensured sufficient humidity for the honey bees. The vials were transferred to an oven set to 43 °C, and survival was recorded by inspecting the vials every 30 min. Three sets of exposures each with 10 animals per exposure group were carried out.

### Statistical analyses

The statistical interface R v.3.4.3 was used for all statistical analyses. The effects of diesel exhaust pollution on the proportion of learned and memorised responses to the odour stimulus were analysed with beta regression models using the *betareg* package^50^. The dependent variables were transformed using the formula y’ = (*y* · (*n* − 1) + 0.5)/*n*), where *y* is the transformed variable and *n* is the sample size, to account for zero- and one-inflation^51^. For the memory trial, the responses of those individuals still surviving at each time point were included in the analyses. The effects of diesel and exposure time on HSP70 expression were analysed using a general linear model. Correlations between exposure time and HSP70 protein expression for diesel exhaust-exposed and unexposed individuals were determined using the function *cor.test* in the package *stats*. Beta regression models were used to analyse the proportion of surviving bees in response to acute diesel exposure and after heat stress over time. Models included air treatment (i.e. diesel and control) and time as fixed terms, as well as the interaction between these terms. Post-hoc tests were performed using the function *lsmeans* in the package *multcomp*^52^.

## Supplementary information

Suplementary Information

## Data Availability

Data from this paper are available at the Dryad digital repository; 10.5061/dryad.vb27ks3.
