# Supplementary material for: Acute exposure to diesel exhaust induces central nervous system stress and altered learning and memory in honey bees
Source: Sci Rep. 2019 Apr 8;9:5793. doi: 10.1038/s41598-019-41876-w (PMC6453880; doi:10.1038/s41598-019-41876-w)
Supplement: Supplementary file 1 — Suplementary Information [file 41598_2019_41876_MOESM1_ESM.docx]

**Supplementary Information**

**Acute exposure to diesel exhaust induces central nervous system stress and altered learning and memory in honey bees**

Christine M. Reitmayer, James M. W. Ryalls, Emily Farthing, Christopher W. Jackson, Robbie D. Girling, Tracey A. Newman

**Table S1.** Full beta regression and linear model statistics showing the effects of time and air treatment (treatment) on learning, memory, HSP70 expression and survival of honey bees (*Apis mellifera*), as well as the effects of heat stress and pollution on bee survival. Values highlighted in bold indicate significance (*P* < 0.05).

| **Response** | **Fig.** | **Time (or trial)** | | |  | **Treatment** | | |  | **Time × Treatment** | | |
| --- | --- | --- | --- | --- | --- | --- | --- | --- | --- | --- | --- | --- |
|  |  | **Stat.** | ***P*** | **df** |  | **Stat.** | ***P*** | **df** |  | **Stat.** | ***P*** | **df** |
| **% learning**  **(30 min exposure)** | 1a | χ^2^  13.60 | **0.004** | 3,16 |  | χ^2^  0.72 | 0.396 | 1,16 |  | χ^2^  1.80 | 0.614 | 3,16 |
| **% learning**  **(150 min exposure)** | 1b | χ^2^  80.58 | **< 0.001** | 3,48 |  | χ^2^  16.35 | **< 0.001** | 1,48 |  | χ^2^  1.14 | 0.768 | 3,48 |
| **% recall**  **(150 min exposure)** | 1c | χ^2^  8.52 | **0.036** | 3,44 |  | χ^2^  4.35 | **0.037** | 1,44 |  | χ^2^  1.45 | 0.695 | 3,44 |
| **HSP70 expression** | 2 | *F*  23.37 | **< 0.001** | 3,65 |  | *F*  0.20 | 0.659 | 1,65 |  | *F*  10.00 | **< 0.001** | 3,65 |
| **% survival** | 3a | χ^2^  8.41 | **0.038** | 3,16 |  | χ^2^  0.85 | 0.357 | 1,16 |  | χ^2^  1.00 | 0.802 | 3,16 |
| **% survival after heat stress** | 3b | χ^2^  120.73 | **< 0.001** | 3,24 |  | χ^2^  8.77 | **0.003** | 1,24 |  | χ^2^  12.28 | **0.006** | 3,24 |

**Fig. S1.** Experimental set up for acute exposures to diesel exhaust or clean air control (A). Collected returning forager honey bees were placed into the one of the glass exposure chambers (B). The glass exposure chambers possess an inlet for the treatment air as well as a ventilation opening. For the clean air control, air was drawn from a tube placed upwind of the experimental site, using a diaphragm pump and then passed through an activated charcoal filter and a distilled water trap, before entering the clean air control exposure chamber. For diesel exhaust exposures, air was drawn from the exhaust pipe of a diesel generator, using a separate diaphragm pump and passed into the diesel exhaust exposure chamber. Flow rates to both chambers were regulated to 1L/min. Glass exposure chambers were kept in a water bath at 20-25^o^C to maintain a constant air temperature within the chambers.

**
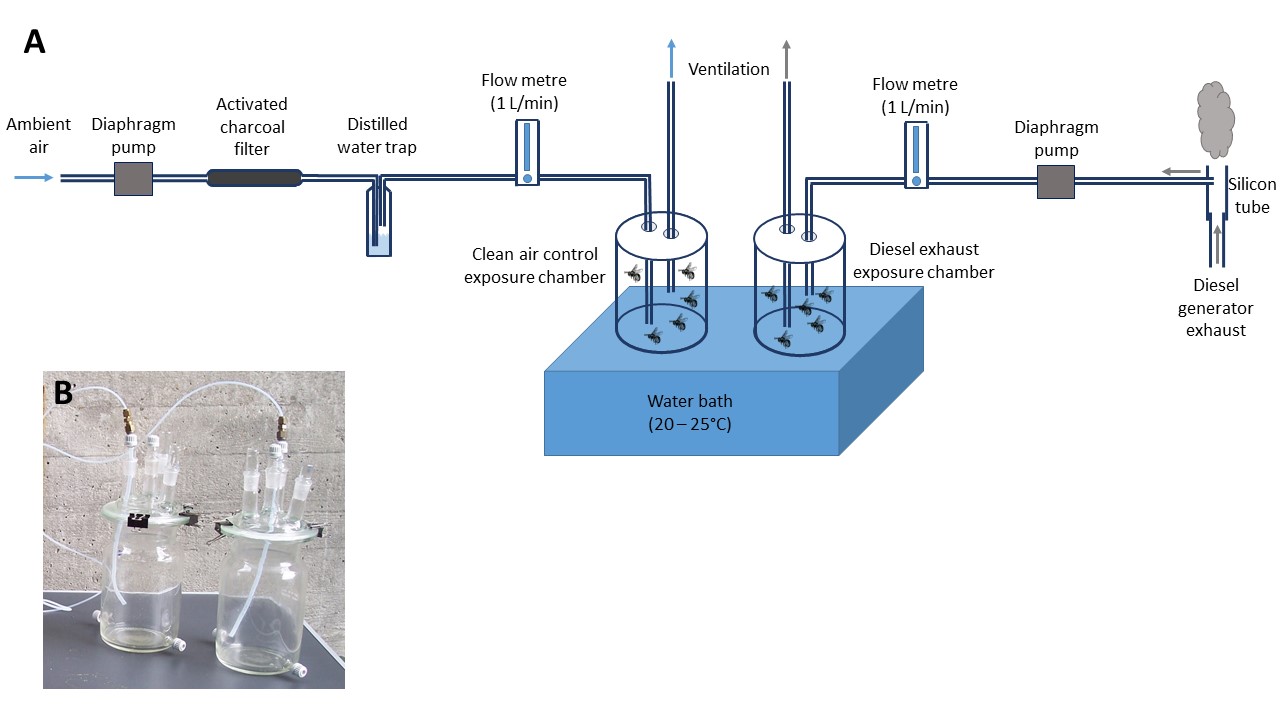
**
